# Genome Sequence of a Novel Alloherpesvirus Isolated from Glass Catfish (Kryptopterus bicirrhis)

**DOI:** 10.1128/genomeA.00403-18

**Published:** 2018-05-10

**Authors:** Rachel N. Henriquez, Jaree Polchana, Somkiat Kanchanakhan, Andrew J. Davison, Thomas B. Waltzek, Kuttichantran Subramaniam

**Affiliations:** a College of Agriculture and Life Sciences, University of Florida, Gainesville, Florida, USA; b Aquatic Animal Health Research and Development Division, Department of Fisheries, Chatuchak, Bangkok, Thailand; c MRC-University of Glasgow Centre for Virus Research, Glasgow, United Kingdom; d Department of Infectious Disease and Immunology, College of Veterinary Medicine, University of Florida, Gainesville, Florida, USA

## Abstract

The 149,343-bp genome of silurid herpesvirus 1, which was isolated in Thailand from glass catfish, was sequenced. The genome was most closely related to that of ictalurid herpesvirus 2, which infects black bullhead catfish. To our knowledge, this was the first silurid catfish alloherpesvirus genome to be sequenced.

## GENOME ANNOUNCEMENT

Viruses in the order Herpesvirales have morphologically characteristic virions consisting of an outer envelope surrounding a proteinaceous matrix termed the tegument, in which is embedded a T = 7 icosahedral capsid enclosing a linear double-stranded DNA genome (1). Viruses in the three families in the order infect mammals, birds, and reptiles (Herpesviridae), fish and amphibians (Alloherpesviridae), and mollusks (Malacoherpesviridae) (2). The family Alloherpesviridae contains four genera, among which the genus Ictalurivirus includes the species Ictalurid herpesvirus *1*, Ictalurid herpesvirus *2*, and Acipenserid herpesvirus *2*. Complete genome sequences are available for ictalurid herpesviruses 1 and 2 (IcHV1 and IcHV2 [3, 4]), which were isolated from channel catfish (Ictalurus punctatus) and black bullhead catfish (Ameiurus melas), respectively.

In 2003, an aquaculture facility in Thailand reported high mortality of unknown etiology in tank-reared glass catfish (Kryptopterus bicirrhis). Moribund fish displayed discolored musculature and abnormal swimming behavior. The viscera of five diseased animals, each about 5 cm in length, were pooled, homogenized, and inoculated onto epithelioma papulosum cyprini cell monolayers grown at 23°C. When cytopathic effect became apparent, DNA was extracted from the clarified cell culture supernatant by using a Maxwell 16 tissue DNA purification kit. A DNA library was prepared by using a Nextera XT kit and sequenced by using a version 3 600-cycle kit on a MiSeq sequencer (Illumina), yielding 4,047,156 quality-filtered paired-end reads.

*De novo* assembly of the reads using SPAdes (5) recovered a large alloherpesvirus-related contig, which was joined to other contigs manually by PCR and Sanger sequencing. The reads were assembled against the resulting contig by using Bowtie 2 (6) and viewed by using Tablet (7). The genome ends were identified from two large sets of reads sharing an end. The reconstructed genome was 149,343 bp in size and consisted of a unique region of 100,111 bp flanked by terminal direct repeats of 24,616 bp. A total of 621,956 (15%) reads matched this sequence at an average coverage of 828 reads per nucleotide. Open reading frames (ORFs) potentially encoding functional proteins were predicted by transferring annotations from the colinear IcHV2 genome (GenBank accession no. MG271984 [4]) using Genome Annotation Transfer Utility (GATU) (8) and by assessing conservation in IcHV2 and IcHV1 (GenBank accession no. M75136 [3]). A total of 94 ORFs were predicted, with 64 ORFs in the unique region and 15 ORFs in each terminal repeat. Each of three ORFs (encoding a really interesting new gene [RING] finger protein, a chloride intracellular channel [CLIC]-like protein, and a membrane-associated protein) was found to be fragmented by an in-frame stop codon, but it was not established when these apparent mutations arose. Phylogenetic analyses performed by using IQ-TREE (9) on the concatenated nucleotide sequences of the second exon of the DNA packaging terminase subunit 1 gene and the entire DNA polymerase catalytic subunit gene showed the closest relationship to IcHV2 (93.2% identity). The International Committee on Taxonomy of Viruses may wish to consider whether a new species (Silurid herpesvirus *1*) should be established for the novel virus.

### Accession number(s).

The silurid herpesvirus 1 genome sequence (strain KRB14001) has been deposited in GenBank under accession no. MH048901.
